# The Developmental Cycle of *Spirodela polyrhiza* Turions: A Model for Turion-Based Duckweed Overwintering?

**DOI:** 10.3390/plants13212993

**Published:** 2024-10-26

**Authors:** Paul Ziegler

**Affiliations:** Department of Plant Physiology, University of Bayreuth, 95440 Bayreuth, Germany; paul.ziegler@uni-bayreuth.de

**Keywords:** duckweeds, Lemnaceae, *Spirodela polyrhiza*, turions, formation, dormancy, germination, sprouting, model organism

## Abstract

Duckweeds are widely distributed small, simply constructed aquatic higher plants (the Lemnaceae) found on quiet freshwater surfaces. Species inhabiting temperate climates may have to cope with long periods of severe cold during the winter season. Several duckweeds form compact resting structures from the assimilatory fronds of the growing season that can bridge inhospitable conditions in a quiescent state. Of these, turions separate from the mother fronds and overwinter on the water body bottom in a dormant state. They can surface, germinate, and sprout to resume active growth upon warming in the spring. The turions of the largest duckweed, *Spirodela polyrhiza*, have been intensively examined as to ultrastructure, the factors governing their formation and release from dormancy, and the signals driving their germination and sprouting and the accompanying starch degradation. Comparative transcriptomics of assimilatory fronds and dormant turions are revealing the molecular features of this developmental cycle. The results illustrate an elegant sequence of reactions that ensures aquatic survival of even severe winters by frost avoidance in a vegetative mode. Since little is known about other duckweed resting fronds, the *S. polyrhiza* turion developmental cycle cannot be considered to be representative of duckweed resting fronds in general but can serve as a reference for corresponding investigations.

## 1. Introduction

Duckweeds are small vascular aquatic higher plants, or macrophytes, that can be found on or just below the surface of quiet fresh water in most parts of the world [1,2,3]. They consist primarily of leaf-like assimilatory organs, or fronds, which are thallus-like structures of from less than 1 to 15 mm in diameter or length and only a few cells in thickness. This represents a fusion of leaves and stems and thus the extreme reduction of an entire vascular plant. During the growing season, the fronds consist largely of spongy mesophyll with extensive air-filled intercellular spaces that confer buoyancy, and the flattened thali of the larger species bear one to several simple hairless adventitious roots on the underside [2,4]. The duckweeds are thought by some to represent a subfamily of the Araceae (the Lemnoideae: see [5]), but by others to constitute a family, the Lemnaceae, in its own right [6]. Until recently, the Lemnaceae have been thought to consist of 36 species [7,8] distributed among the five genera *Spirodela* (abbreviation *S.*), *Landoltia* (*La.*), *Lemna* (*Le.*), *Wolffiella* (*Wa.*), and *Wolffia* (*Wo.*). However, it has been shown that the putative species *Le. japonica* is actually a hybrid of the two authentic species *Le. minor* and *Le. turionifera* (correct nomenclature thus *Lemna x japonica*: [9]), and that *Le. minor* also forms an interspecific hybrid with *Le. gibba* that exists in nature (*Le. x mediterranea*: [10,11,12]. The 35 species and two interspecific hybrids exhibit genus-typical differences in the size and complexity of the fronds and in the number of roots they bear [2,3,4,13,14,15,16,17]: see also the figure presented in Section 2.2. These differences reflect an evolutionary progression from *Spirodela* to *Wolffia* featuring morphological reduction and genome augmentation [4,18]. The genus *Wolffia* includes the world’s smallest flowering plants [2,19].

Duckweeds grow by reproducing the fronds characteristic of the growing season. Daughter fronds bud off vegetatively from one or two meristematic pouches or pockets in the mother fronds and give rise to subsequent daughter fronds. The fronds remain attached to one another for a time after their formation via stipes to give rise to colonies of 2 to 50 connected fronds [2,4,20,21,22]. The colonies of interconnected fronds spread out over the water surface to ensure optimal access to water nutrients [13]. Duckweed growth thus constitutes clonal replication and manifests itself in an increase in frond number, as well as in an increase in frond area or weight. It can take place very rapidly under favorable conditions: duckweeds have been shown to include the most rapidly growing higher plants [23,24].

Duckweeds inhabit all climate zones except the very cold polar regions and extremely dry desserts. Cosmopolitan species can be found on all continents and under a wide range of climatic conditions, whereas the occurrence of other species is confined to more limited geographical regions and climates [2,3,15,25]. The success of duckweeds in colonizing compatible water bodies and persisting in these habitats is a function of the small size of the macrophytes in conjunction with the ability to grow very rapidly, the vegetative clonal propagation scheme, and a neotenous or juvenile organizational status that facilitates the formation of advantageous frond derivatives when appropriate. A recent publication describes how these attributes enable duckweeds to establish themselves in new locations and to productively respond to environmental challenges including problems posed by temperature, light, pH, mineral supply, microbial attack, water pollution, competition, and drought [25]. Duckweeds that inhabit regions with cold winters must be able to cope with low temperatures that may fall well below the freezing point. Plant material can survive cold by tolerating the low temperatures involved, or by avoiding exposure to them if they are life-threatening. Duckweed growth does not usually take place at temperatures lower than 8–17 °C, but fronds can often tolerate temperatures down to the freezing point for some period of time [2,26]. However, duckweed fronds are thought to usually not be able to withstand prolonged or severe frost [2,25,26]. There are reports of duckweed fronds surviving encasement in ice (and thus possible exposure to very low temperatures) for an extended period ([26] respective of *Le. minor*; [27] respective of *S. polyrhiza*), but these observations have not been followed up upon. Duckweeds thus rather cope with seasonal cold by tolerating temperatures down to and not significantly below the freezing point and by avoiding exposure to severe frost. They do this via the formation of ***resting fronds*** upon the onset of winter conditions as modifications of the actively proliferating fronds produced during the growing season. In the following Section 2, the features of the different types of resting fronds are discussed to illustrate how these structures enable duckweeds to survive cold to very cold winters.

## 2. Resting Fronds

Resting fronds are frond derivatives produced by the meristematic pockets of fronds typical of the growing season (these actively metabolizing structures will be referred to as “normal fronds” or simply “fronds” in the following) in response to environmental influences heralding the onset of the winter season. They are generally smaller and more robust than “normal” fronds, with fewer air spaces and higher starch contents [2,4,13,26]. They exhibit reduced or completely arrested growth and corresponding low metabolic activity at low temperatures that enable them to subsist under conditions unfavorable for growth and propagation. Resting fronds can resume vigorous growth and give rise to new growing fronds when temperatures rise again sufficiently. The production of resting fronds and their subsequent “activation” enable the survival of cold periods in a vegetative mode. Two principal types of resting fronds can develop:

### 2.1. Resting Fronds Still Capable of Growth

Some resting fronds are similar in appearance to normal fronds, although thicker and fleshier in appearance. Their metabolic potential is strongly inhibited due to internal constraints during the winter season, but they can still grow and even reproduce slowly when the cold is not too severe [2]. Their metabolic inactivity at low temperatures is a form of “quiescence” due to external constraints rather than the true or innate “dormancy” to be described in the following. They can give rise to new fronds when conditions improve.

*La. punctata*, *Le. perpusilla*, *Le. gibba*, *Le. minor*, most strains of *Le. aequinoctialis*, and some strains of *Le. japonica* (now *Le. x japonica*: see [9] and the Introduction above) form resting fronds capable of growth that remain on the water surface [2]. This is suitable for survival only at temperatures down to and not significantly lower than the freezing point, i.e., cold, but not freezing water, which can be tolerated without lethal effects. These resting fronds may, however, also avoid the effects of severe frost by being pressed beneath ice forming on the water surface or by remaining attached via stipes to the pouches of mother fronds that have died and sunk to the bottom of the water body [2]. *Le. minor* resting fronds, which are generally thought to overwinter on the water surface, survived very cold winters beneath massive ice layers on a pond in Quebec, Canada [28].

*Le. trisulca*, *Wa. gladiata*, and *Wo. arrhiza* form resting fronds capable of growth that sink to the bottom of the water body due to their density occasioned by reduced air spaces and high starch content [2]. Since water temperatures on the bottom hardly go below the freezing point, the resting fronds avoid severe frost temperatures that may be in effect at the water surface in their submerged surroundings. These resting fronds thus provide for survival in even very cold winters.

### 2.2. Turions

Turions are resting fronds that are smaller than and morphologically distinct from the mother fronds having given rise to them; they separate from the mother fronds, sink to the bottom of the body of water inhabited by the mother fronds and do not grow further [2]. According to Landolt [2], they are found in *S. polyrhiza*, *Le. turionifera*, some clones of *Le. aequinoctialis*, and many species of *Wolffia* (*Wo. brasiliensis*, *Wo. borealis*, Wo. *angusta*, *Wo. australiana*, *Wo. arrhiza*, *Wo. columbiana*, and *Wo. globosa*). They have also been reported to occur in *Wo. cylindracea* and *Wo. neglecta* [29] and in *Wa. floridana* (now *Wa. gladiata*: see [2]) [30] and have been observed in *Wo. microscopica* [25]. The turions of *S. polyrhiza* and *Le. turionifera* are flat and rounded, while those of *Wolffia* are very small and spherical [2]. Turions characteristically have thicker cell walls, much smaller air spaces and vacuoles, and much higher starch contents than the “normal” fronds giving rise to them. They also exhibit only very rudimentary roots, closed stomata, and high anthocyanin contents [2].

**Figure 1:**
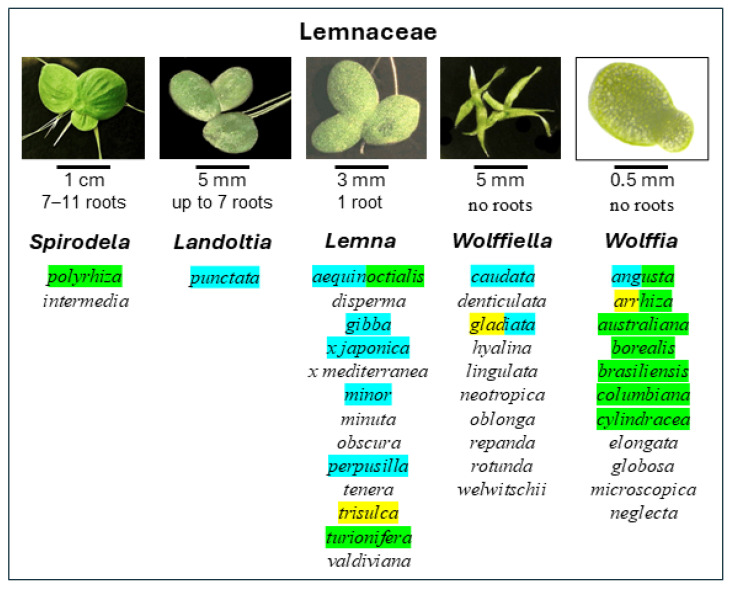
The five genera of the Lemnaceae and their respective species, including the two interspecific hybrids *Le. x japonica* and *Le. x mediterranea*. Species reported to form resting fronds capable of growth that remain on the water surface as described in Section 2. are marked with **blue**, those reported to form resting fronds capable of growth that sink to the bottom of the water body are marked with **yellow**, and those reported to form true turions are marked with **green**. The images are of *S. polyrhiza* strain 9500, *La punctata* 5562, *Le. minor* 9441, *Wa. gladiata* 7173, and *Wo. arrhiza* 9528, all courtesy of Klaus Appenroth.

An overview of the duckweed species having been reported to form the different types of resting fronds is presented in Figure 1.

Whereas some turions may be as sensitive to cold as are the corresponding fronds of the species (e.g., *Wo. arrhiza*: [31]), others such as those of *S. polyrhiza* are more tolerant of low temperatures than are the corresponding fronds [2] (*S. polyrhiza* fronds have been described as being “frost-sensitive” [29]). However, no turions are known to be able to tolerate prolonged, severe frost [2], and all must be able to avoid direct exposure to intense cold in order to survive through prolonged, severe winter conditions. They do this at the bottom of the water body to which they sink beneath surface ice where the water temperatures fall scarcely below the freezing point. In this manner, they resemble the submerged resting fronds that are still capable of growth.

Since resting fronds share some structural and functional equivalence with turions, they are often described as being “turion-like” resting fronds (e.g., [26]). True turions are, however, truly, or innately, dormant upon their formation. Innate dormancy can be regarded as a state of developmental intermission in which growth and other developmental processes are arrested by certain internal conditions [26]. Turions thus do not—and cannot—grow for some time after their formation, although they may exhibit some respiration and are capable of photosynthesis (e.g., [32]). Duckweed turions become capable of resuming growth once more after a prolonged period of exposure to low but not freezing temperatures. This “***after-ripening***” (turion formation can be termed “ripening”) breaks the dormancy and allows the turion to germinate and sprout to form new actively growing fronds when conditions again become conducive to growth and propagation [33]. Turion dormancy will be discussed in more detail in Section 3.2. Duckweed turions are particular examples of detachable, truly dormant modified green shoots that function as vegetative propagules in many aquatic plants [1,2,34,35]. Turions are found in 11 genera of aquatic vascular plants other than duckweeds [35].

The readily evident ecological context of the seasonal developmental cycle of duckweed turions—formation in response to approaching winter, waiting out the winter in a dormant state removed from severe frost, and resumption of growth and propagation when the winter has passed—has long stimulated interest in the characterization and understanding of the developmental processes involved. While turions have been reported to occur in 14 duckweed species distributed among three genera (see above), it is remarkable that by far the most investigations of duckweed turions have been carried out on a single species: *S. polyrhiza*, the largest of the duckweeds and often referred to as the Great, Greater or Giant Duckweed. The work done on *S. polyrhiza* provides a comprehensive picture of how the turions of a particular duckweed are formed, rest in a dormant state, and then subsequently germinate and sprout to resume growth in the context of approaching winter, winter itself, and the passing of winter. Two early extensive investigations dealing specifically with the *S. polyrhiza* turion developmental cycle were published by Jacobs [36] and Henssen [37], and the relevant knowledge available up till the middle of the 1980s was compiled and evaluated by Landolt [2] and Landolt & Kandeler [26]. In the following, the acquisition of our present knowledge as to the main features of the developmental cycle of *S. polyrhiza* turions—turion formation, dormancy, and activation as germination and sprouting—is traced in Section 3. The issue of whether our present concept of the workings of this cycle justifies the regarding of *S. polyrhiza* as a model organism for understanding turion- (or even overall resting frond-) based duckweed overwintering is then examined in Section 4.

## 3. The Developmental Cycle of *Spirodela polyrhiza* Turions

**Figure 2:**
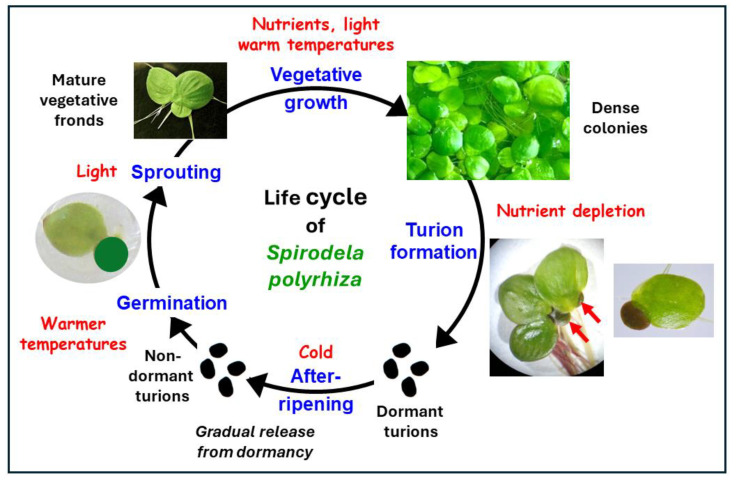
The life, or developmental, cycle of *Spirodela polyrhiza*. The main features of the cycle are designated in **blue**, and important principles governing these features, which are discussed further in the following Section 3.1, Section 3.2 and Section 3.3 are indicated in **red**. The images are of *S. polyrhiza* strain 9500, courtesy of Klaus Appenroth. Turions emerging from a colony of fronds are indicated by red arrows.

The turion-based life cycle of *Spirodela polyrhiza* is depicted in Figure 2. Our knowledge concerning the major features of the turion developmental cycle—the formation, dormancy, and subsequent activation of the turions resulting in the resumption of growth—is presented in the following Section 3.1, Section 3.2 and Section 3.3. The cycle begins with the formation of turions from the “normal” fronds of the growing season.

### 3.1. Turion Formation

When turions are formed in duckweeds, there is a switch in the developmental program of the primordia of “normal” fronds characteristic of the growing season from the formation of new “normal” fronds to the production of resting derivatives [38]. This switch is initiated by environmental signals that exert themselves upon the approach of conditions that are unfavorable to continued growth. The impact of these signals sets a series of molecular and biochemical events in operation—a transduction chain—that results in the formation of the characteristic structure and physiological and molecular makeup of the turion.

Duckweed species exhibit pronounced clonal diversity in characteristics such as their growth potential [24] and their ability to tolerate salinity [39] and to accumulate starch under nutrient deficiency [40]; clonal differences can be genotyped [41]. Turion formation in *S. polyrhiza* also shows great clonal variation when expressed as the specific turion yield SY or number of turions formed per frond [42,43], which is a determinant of the number of turions available to support the survival of the duckweed in winter [38]. Variability in SY represents adaptations to local climatic conditions and is presumably genetically determined [43]. The mean annual temperature of a site inhabited by a *S. polyrhiza* clone has the most important influence on the SY of that clone. Low temperatures result in increased SY to offset the reduced survival rate of the turions under these conditions [43]. In this regard, *S. polyrhiza* accessions from northern latitudes tend to produce turions earlier than those from more southern latitudes under warm experimental conditions [44]. Clonal differences in turion formation such as SY are independent of the specific signals that induce turion formation and are located in the transduction chain leading to the developmental switch from “normal” frond replication to turion production [38]. This also applies to clonal differences in the time required for turions to be formed in response to inductive signals [38].

#### 3.1.1. Signals Governing Turion Formation

The formation of turions in temperate, winter-cold climates upon the advent of the cold season might be expected to be initiated by shortening day lengths and/or decreasing temperatures that herald the onset of winter. In *S. polyrhiza*, however, a shortage of mineral ions such as nitrate, sulphate, and especially phosphate in the medium has been found to result in turion production (see [45]) and to constitute the prime environmental factor in effecting turion formation [46,47]. Turion formation in *S. polyrhiza* was found to be induced mainly by the external phosphate concentration below a defined, clone-dependent threshhold [42]. The exhaustion of water mineral resources resulting from profuse plant growth having taken place throughout the summer is thus the main factor responsible for turion production in *S. polyrhiza* in the autumn. The developmental switch from the continued production of “normal” growing fronds to turion formation is also brought about to a certain degree by low temperatures when the mineral ion nutrition is sufficient [46,47]. The decreasing temperatures of autumn thus represent a “backup” factor ensuring turion formation prior to the onset of winter even when water nutrients have not been significantly depleted. Exogenous abscisic acid (ABA) also induces turion formation in *S. polyrhiza*, as is discussed below. The observation that turion formation was induced in *S. polyrhiza* fronds treated with cadmium [48] or chromate [49] indicates that turion formation may also be a response to some types of water contamination in nature. In a related vein, substances produced by the presence of the water soldier *Stratiotes aloides* were observed to induce turion production in *S. polyrhiza* [50].

*S. polyrhiza* can form turions in darkness as well as in light in the presence of sufficient carbohydrate under conditions of mineral salt deficiency [51]. Exogenously added sugars, as well as high light intensity and CO_2_ concentrations, can enhance turion formation in *S. polyrhiza* once this has been induced. This is due to an increment in turion-producing capacity or the total yield (TY) of turions formed by a frond system, rather than representing a switch in the developmental program of the frond primordia, which is evaluated in terms of the specific turion yield (SY) [38,46,47]. This can be seen in the context of turion formation occurring at the expense of “normal” frond growth, which ceases upon the production of turions. The growth-inhibited mother fronds producing turions must nevertheless have sufficient carbohydrate at their disposal for turion formation while being inhibited in their “normal” growth (see [2]). The more carbohydrates a system of mother fronds can produce—or assimilate—due to better conditions for photosynthesis or to sugar supply, the more turion biomass it can produce. In nature, light intensity remains sufficient to maintain photosynthetic carbohydrate production well into autumn, while low temperature and nutrient deficiency reduce growth without slowing photosynthesis to the same extent [40,52].

Photomorphogenic effects of light via phytochrome involvement can modulate turion formation in *S. polyrhiza* [53], but no critical day length and thus no inductive effect of photoperiod has been observed with this species [54]. It is remarkable that short days, which also herald the onset of the winter season, do not induce turion formation in *S. polyrhiza*. Decreasing mineral nutrient availability combined with decreasing temperatures (especially during the nights) thus give rise to *S. polyrhiza* turion formation in nature in place of the low temperatures and short photoperiods usually responsible for turion formation in other hydrophytes [34,35,38,54]. Irrespective of the inductive conditions, turions of *S. polyrhiza* are produced earlier by late birth order than by early birth order parent fronds [55].

Some environmental influences are detrimental to turion formation. The presence of nickel inhibited turion formation in *S. polyrhiza*, whereupon the carbohydrate content in the mother fronds, the growth of which was also inhibited, increased. It was assumed that nickel inhibited the transport of photoassimilate from the mother fronds to the developing turions [56]. Other environmental signals inhibit turion formation without overly affecting growth of the mother fronds. Temperatures high enough to be only slightly inhibitory to frond growth blocked turion formation in *S. polyrhiza* completely, and salinity inhibited turion formation much more severely than it did frond growth in this species [57].

#### 3.1.2. The Molecular Nature of Turion Formation

Only stimuli that initiate the reprogramming of the developmental program of the frond primordia from continued “normal” frond production to the formation of resting derivatives are of importance for the understanding of the molecular basis of turion formation. As mentioned above, these include nutrient salt depletion, low temperature, and water contaminants such as cadmium, in contrast to high light intensity, high CO_2_ concentration, or external sugar addition that only enhance the extent of turion formation once this has been initiated. How these stimuli initiate turion formation is not easily evident. A useful tool in this regard has been the external application of the plant hormone abscisic acid (ABA), which has long been known to regulate plant growth, development, and stress responses [58]. Exogenous ABA was early found to both inhibit frond growth and initiate turion production in *S. polyrhiza* [59,60], thus effecting a true switch in the developmental program of the frond primordia of *S. polyrhiza* equivalent to that caused by nutrient deficiency and low temperatures [46]. This phytohormone has played an important role in the investigation of the processes leading to turion formation. Although exogenously applied ABA is not a natural environmental signal, the release of ABA from an old culture of *S. polyrhiza* into the medium of a fresh culture has been reported to correlate with the onset of turion formation in the fresh culture [61].

##### The Turion-Inducing Effect of Abscisic Acid

Externally added ABA induced turion formation in *S. polyrhiza* best at a concentration of 0.1 mM, at which only frond primordia ≤0.7 mm in length at the time of ABA addition developed into turions. This corresponds to a 14–20 h window of sensitivity to ABA in the frond primordia. Primordia cells perceiving ABA within the sensitivity window initiate the formation of turions instead of continually producing new “normal” fronds [62]. ABA treatment of *S. polyrhiza* leading to turion formation corresponded to a physiological increase in the tissue ABA level from 75 nM to about 1 µM, allowing an estimate of actual endogenous ABA concentration changes associated with turion formation induction. This indicated that ABA has a physiological role in turion formation [63], a role now considered to be confirmed (see [64]). It was also proposed that ABA interacts with a plasmalemma receptor system to induce turion formation [63]. These studies were integral to the concept of *S. polyrhiza* turion formation as a model system for investigating the molecular action of ABA and dormant bud induction [65,66] (current reviews of ABA and bud dormancy are confined rather to perennial plants (e.g., [67])). ABA synthesis has been observed in terrestrial plant leaves and roots in response to environmental stressors such as salinity and dehydration; it then controls downstream responses via both transcriptional and posttranscriptional mechanisms [58]. If endogenous ABA concentration changes are indeed a decisive factor in S. *polyrhiza* turion formation, it will be useful to determine how low temperatures and especially phosphate limitation result in these changes.

##### Specific Molecular Events Associated with Turion Formation

The use of ABA as a simple reliable trigger to induce turion formation in *S. polyrhiza* [66] has facilitated the examination of the molecular events associated with the induction in this duckweed. Early characterization of ABA-induced turion formation in *S. polyrhiza* illustrated the development of turion-specific ultrastructural features, specific changes in protein synthesis and mRNA profiles, and altered ion fluxes across membranes [68,69,70]. Reduction of the synthesis of the bifunctional enzyme UDP-apiose/UDP-xylose synthase was suggested to underlie a considerably reduced pool of total labelled UDP-apiose derivatives in ABA-induced turions in comparison with the mother fronds [71]. ABA-induced turion formation resulted in an up-regulation of mRNA transcripts of a gene homologous to D-myo-inositol-3-phosphate synthase (designated “Tur1”) localized to stolon tissue connecting the developing turion to the meristematic region of the mother frond [72]. An increase in the levels of free inositol and inositol phosphates as potential turion storage compounds were also shown to accompany the ABA-induced turion formation [73]. Turion formation induced by ABA resulted in a transient, plant-wide expression enhancement of a gene homologous to a yeast ABC (ATP-binding cassette) transporter (“Tur2”) that is otherwise induced by environmental stress treatments [74]. ABA-induced turion formation also led to a transient increase in the level of mRNA transcripts encoding a basic peroxidase (“Tur4”: [75]).

The main storage compound of *S. polyrhiza* turions is the starch that imparts the propagules with their density and constitutes most of the reserve material for their overwintering and subsequent germination and sprouting. Wang and Messing [76] cloned and sequenced three different genes of the large subunit of the ADP-glucose pyrophosphorylase (AGPase) of *S. polyrhiza*. AGPase is the gateway enzyme of starch synthesis [77]; its large subunits are responsible for the allosteric regulation of the catalytic activity (see [78]). In showing that 14 days of exposure of *S. polyrhiza* fronds to 1 µM ABA resulted in the production of turions exhibiting a starch content of 60% of dry weight (DW), the three *SpAPL* genes were differentially expressed throughout the growth of fronds and the subsequent induction and development of the turions. *SpAPL1* expression declined soon after the ABA addition, while the expression of *SpAPL2* and *SpAPL3* was considerably enhanced during the initiation of the turion formation and the further development of the turions, respectively. The findings, respective of *SpAPL1* and *SpAPL3* were confirmed in a later transcriptome analysis of *S. polyrhiza* ABA-elicited turion development by Wang and co-workers [79], and the recent transcriptomic study of Pasaribu and co-workers [80] revealed *SpAPL2* to be expressed at a far higher rate in mature turions than in “normal” fronds [81]. The results of these transcriptome investigations—which are discussed in more detail in the following—suggest that one effect of ABA in mediating *S. polyrhiza* turion formation is to switch the enzymatic focus of starch formation from “normal” fronds to the propagules being developed from them.

An essential regulatory role in plant growth and stress response is played by growth-regulating factors (GRFs) that are plant-specific transcription factors. Six GRFs were identified in *S. polyrhiza* fronds: of these, SpGRF3 transcripts were significantly higher in turions than in the mother fronds having given rise to them. Expression analysis during ABA-induced turion formation showed that the levels of the transcripts of all six GRFs in the turion-producing fronds decreased during the first three days of ABA treatment, then gradually increased to decrease again at 14 days of treatment. These results were interpreted to suggest that SGRF3 may be a negative regulator of leaf size in the duckweed and thus responsible for the smaller size of the turion [82].

##### Transcriptome Analyses of *S. polyrhiza* Turion Formation

A transcriptome analysis of ABA-triggered turion development in *S. polyrhiza* carried out by Wang and co-workers in 2014 [79] gave a first indication of the extent of differential gene expression accompanying turion formation. Fronds were grown in the presence of 10 µM ABA for three days, at which time they were irreversibly committed to the formation of turions. They then consisted of growth-inhibited mother fronds producing turions in the first stages of development: turions were mature 14 days after being exposed to ABA. RNA was isolated from control fronds and from the 3-day ABA-treated fronds, sequenced and mapped to a reference *S. polyrhiza* genome. A total of 362 genes in the ABA-treated fronds with developing turions were observed to differ to an extent of more than 4-fold in comparison with the control fronds, whereby 208 of the differently expressed genes (DE-genes) increased in expression and 154 decreased. The up-regulated genes included those involved in signal transduction and in carbohydrate and secondary metabolism and senescence. A total of 25 of the up-regulated DE genes belonged to gene families involved in the response to ABA stimulus and to the negative regulation of ABA-mediated signalling pathways, illustrating the complexity of the action of this phytohormone in turion development. The up-regulation of three genes encoding late embryogenesis-abundant (LEA) proteins that act as protectants for proteins in seeds was considered to be a good marker for the dormancy of the developing turions. Transcription of the APL1 and APL3 genes (but not of the APL2 gene) encoding AGPase was significantly increased as had previously been observed ([76]: see above), as was that of genes encoding a granule-bound starch synthase (GBSS1) and the basic peroxidase “Tur4” also having previously been described [75]. Repressed genes included those responsible for rapid growth and biomass accumulation, protein synthesis, and carbon fixation. It was shortly thereafter deduced from enhanced methylation of *S. polyrhiza* DNA upon ABA-induced turion formation that the phytohormone might affect gene expression by altering the methylation status of the cytosine nucleotide [83]. DNA methylation could thus have been involved in the differential gene expression described by Wang and co-workers [79].

The transcriptional studies of (ABA-induced) *S. polyrhiza* turion formation described above were problematic in two major respects. Since the sample material representative of turion formation consisted of fronds that were in the process of producing turions, RNA isolated from the developing turions was not free of that originating from the corresponding mother frond tissues and was as such not exclusively representative of the turion RNA. The RNA contents of fully formed, mature turions were not investigated (only the ultrastructure of such turions had been examined [79]), because the nucleic acids could not be satisfactorily extracted from the propagules due to their thick cell walls and large contents of starch, anthocyanins and tannins (see [80,81]). To remedy these shortcomings, Pasaribu and co-workers [80] harvested mature turions produced by and separated from *S. polyrhiza* fronds and extracted nucleic acids from them after homogenizing the tissues by a novel combination of grinding in liquid nitrogen and vigorous shaking with silica beads. The formation of the turions was induced by phosphate deprivation (and thus by the principal natural stimulus instead of by artificial ABA treatment) of the fronds of a *S. polyrhiza* clone (sp9512) characterized by particularly rapid propagule formation. A high-quality de novo sp9512 genome assembly was generated from the fronds and characterized, and high-quality reads of sequenced RNA isolated from the fronds and turions were mapped to the p9512 genome. This strategy yielded high-quality transcriptome libraries from both fronds and mature turions, enabling the comprehensive description and comparison of actively growing frond and dormant turion transcriptomes [80,81].

The analysis of Pasaribu and co-workers [80] detected transcripts in the combined frond and turion transcriptome libraries representing almost 95% of the 18,403 protein-encoding genes annotated in the sp9512 genome. The expression of 81% of the genes corresponding to these transcripts changed to an extent of less than a 4-fold the course of the formation and maturation of the turions. The transcript levels of about of 80% of the expressed *S. polyrhiza* genes accordingly did not differ to a significant extent between the developmental states of frond and mature turion, and the transcript abundance of most of the expressed genes of the dormant turions is thus similar to that of the metabolically active frond tissues. Of the remaining 3260 genes showing greater expression differences between fronds and turions, numerous examples representing four major categories of physiological pathways were overexpressed by 8-fold or more in turions in comparison to frond tissues. Strongly reduced (≥8-fold) gene expression in turions was observed for several physiological categories of universal functional importance for actively growing fronds. The transcripts in the frond extracts were indicative of ongoing translation and constituted a snapshot of current metabolic activity. The transcripts of the turions were not translated due to the metabolic inactivity of the dormant propagules and constitute a depot of translational potential that can first be realized when the dormancy of the turions is broken and metabolic activity sets in.

The four physiological categories corresponding to increased transcription in *S. polyrhiza* turions were stress responses, secondary metabolite and lipid metabolism, defense responses, and seed development and germination [80]. Many up-regulated genes relating to ABA signaling and synthesis in the first, second and fourth categories illustrate the importance of ABA-mediated pathways in turion formation. The second category also featured the upregulation of genes encoding both oleosins (proteins that protect the surface of lipid droplets from lipase attack—and lipases that effect lipid degradation. It also encompasses genes related to starch metabolism. The upregulation of the APL2 and APL3 members of AGPase during turion formation concurrent to the downregulation of the APL1 transcript level of the fronds illustrates the switch in starch synthesis from fronds to turion in conformation with the massive starch accumulation in the latter, while confirming and complementing the corresponding findings of Wang and co-workers [76,79]. At the same time, the expression of a chloroplastic α-glucan, water dikinase that mediates starch degradation was far higher in turions than in fronds. The enhanced expression of several defense-related enzymes and proteins in the third category indicates that turions are well-equipped to repel attack by pathogens such as bacteria, fungi, and herbivores. Differentially expressed genes of the fourth category include several that resemble those well-known to mediate seed development and embryo formation in plants exhibiting sexual reproduction, as well as some thought to be important in germination physiology. Examples are the activation during turion formation of genes encoding LEA proteins that act as cellular protectants during seed formation and the up-regulation of enzymes known to play a role in lipid degradation.

Strongly reduced (≥8-fold) gene expression in mature turions was related to several physiological categories of universal functional importance for actively growing fronds [80]. Transcripts related to cell division, DNA replication, cytoskeleton-related processes, organelle fission, photomorphogenesis, and ion transport were significantly suppressed during turion maturation. This also applied to the gene families of RubisCo and light-harvesting proteins that are particularly highly expressed in photosynthetically active fronds. Significantly different contents of some cellular components and compounds were found between fronds and turions. A significantly lower level of mitochondrial DNA content was observed in mature turions, indicating a decreased need for this organelle in these propagules. Turions had considerably lower contents of free fatty acids and saturated triacylglycerol (TAG) fatty acids than fronds, while having higher contents of TAG and the di-unsaturated linoleic TAG fatty acid. Global and specific increases in cytosine methylation content provided indications that epigenetic modification played a role in the turion formation.

The differential expression of specific *S. polyrhiza* genes observed between the fronds and mature turions [80] largely confirmed many of the transcriptional effects observed as described above respective of ABA-induced turion formation. This refers on the one hand to the probable responsibility of a combination of UDP-apiose and UDP-xylose synthase activities for the low levels of UDP-apiose found in *S. polyrhiza* turions [71]. It also applies to the increased expression and stolon localization of Tur1 [72], although Tur1 was found to show enhanced expression in the turions as well─possibly for the synthesis of phytate as a phosphate storage compound─and **a** second ortholog to Tur1 was found to be more active in fronds than in turions. The transient increases in both Tur2 and Tur 4 expression in turions reported by [74,75] were also observed again, albeit not to significant extents, as well as the lack of differentiation of Tur 2 expression between fronds and turions. Altogether, Tur 2 and Tur 4 expression was not indicated to be specifically related to turion formation. It is noteworthy that the study of Pasaribu and co-workers [80] also confirmed the differential expression of the AGPase genes APL1-3 reported by Wang and Messing [76], the increased expression of LEA proteins in turions found by Wang and co-workers [79] and the probable role of cytosine methylation in the epigenetic regulation of differential genes expression in *S. polyrhiza* fronds and turions suggested by Zhao and co-workers [83].

It should be emphasized that the transcriptome analysis of Pasaribu and co-workers [80] compares mature, dormant turions of *S. polyrhiza* with the metabolically active fronds that gave rise to them. Whereas the transcripts of the fronds are indicative of ongoing active metabolism prior to the commencement of turion formation, those of the turions define the final, arrested state or product of a formation process. The differentiation of the results between the turions and the fronds shows *what has* happened, but not *how* it happened. This latter is quite different to the transcriptional status of the samples analyzed by Wang and co-workers [79] three days after the application of ABA to the fronds. While these fronds were now committed to the formation of turions, the propagules being formed were still far from maturation. The transcripts of these samples thus represent both fronds pursuing a developmental program different to that in effect prior to ABA stimulus and the structures developing as a result of this altered programming. They accordingly truly reflect turion formation itself (*what* is happening) in contrast to the final result of the formation process. A combination of the two approaches—incorporating samples taken at multiple stages along the road to turion formation—could illustrate more comprehensively how turion formation proceeds to its completion.

#### 3.1.3. The Final Product: The Turion

**Figure 3:**
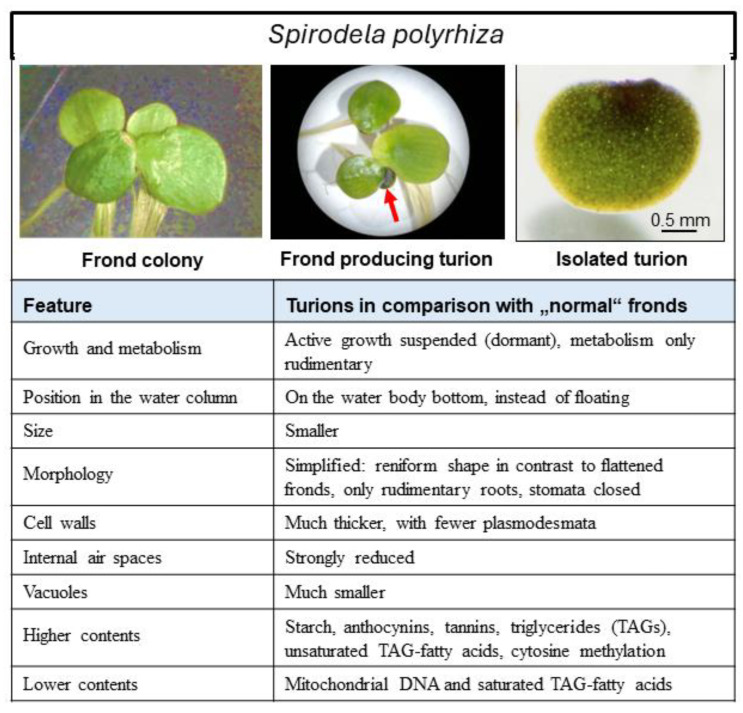
Features of *S. polyrhiza* turions that distinguish them from the vegetative fronds having given rise to them. Images are of *S. polyrhiza* strain 9500, courtesy of Klaus Appenroth. The red arrow points to a turion emerging from a mother frond.

The signaling that initiates turion formation from *S. polyrhiza* fronds and the differential gene expression deriving from it results in the compact, reniform-shaped mature turions of this species. These vegetative propagules have thicker cell walls with fewer plasmodesmata, much smaller air spaces and vacuoles, higher contents of anthocyanins, tannins, and TAGs, and much more starch than the mother fronds giving rise to them [2,14,36,68,79,80,84,85]. They also possess only rudimentary roots. The newly formed turions separate from the mother frond by means of a break at a primary abscjssion layer at the turion base of the short stolon connecting the turion with the mother frond [20,21]. These distinguishing features are summarized in Figure 3 The thick cell walls, small air spaces, and high starch content of the turions cause the propagules to sink to the bottom of the water body on which their mother fronds had grown and to remain there until they surface in the spring (see Section 3.3.1.).

The robust *S. polyrhiza* turion is physically well-suited for spending long periods submerged in cold surroundings. Its dormancy (which is discussed in detail in Section 3.2.) helps it endure lengthy periods under unfavorable conditions in a quiescent state that requires very little energy expenditure. When dormancy ends and conditions permit, the turion can germinate and resume active frond growth (see Section 3.3.). For this purpose, it possesses two meristematic pockets containing primordia from which the new vegetative fronds can develop [84]. The turion is a storage organ that has accumulated large amounts of starch as well as of TAGs that can be mobilized to support frond growth once dormancy has ended and germination has taken place. Indeed, the high starch content of *S. polyrhiza* turions is regarded as a promising feedstock for biofuel production [86], and corresponding efforts are being made to optimize culture methods for turion and starch production with this duckweed (e.g., [87]). The storage function of *S. polyrhiza* turions also includes the presence of most of the transcripts of frond-expressed (“housekeeping”) genes in the turions at levels similar to those in fronds and the heightened expression of transcripts for starch and lipid turnover [80,81]. Since these transcripts are not translated due to the dormancy of the turion, they can be “on hold” for immediate use when significant metabolism sets in upon germination and sprouting. The gene expression described by Pasaribu and co-workers as characteristic of *S. polyriza* turions [80] thus defines the physiological potential of the dormant propagule and lays the groundwork for the resumption of growth activity under appropriate conditions. In a similar vein, photosynthetic pigments maintained in the turions, although present in considerably lower contents than in “normal” fronds, ensure a good photosynthetic capacity to support sprouting after germination takes place [88].

### 3.2. Turion Dormancy

Turions of *S. polyrhiza* produced under natural conditions are unable to resume growth under any naturally prevailing conditions for a protracted period after their formation. The structure of *S. polyrhiza* turions having been revealed by microscopic investigation (see Section 3.1.3.) itself reflects a resting state: the thick cell walls with strongly reduced plasmodesmata [14], small air spaces, and starch-filled plastids are not indicative of active metabolism. The quiescence afforded by the arrest of growth and development that constitutes this innate dormancy enables the turions to endure long periods in cold water at the water body bottom without any appreciable loss of substance [89]. The innate dormancy is eventually lost to give way to imposed dormancy, in which growth and development can resume upon the onset of favorable conditions.

#### 3.2.1. Innate Dormancy

The complete lack of growth of the innate turions precludes any appreciable degree of metabolism and hence of transcription or translation, the latter being evident from the presence of transcripts stored in the newly formed *S. polyrhiza* turions as described by Pasaribu and co-workers [80]. The dormancy of turions represents a metabolic block, or state of “self-arrest” [90] that impedes pre-disposed responses to growth-promoting signals. This condition in *S. polyrhiza* is the summary result of the differential gene expression involved in the turion formation as described by Wang and co-workers [79] and especially by Pasaribu and co-workers [80]. The metabolic block may involve an inhibitory substance—possibly abscisic acid (ABA), which is involved in *S. polyrhiza* turion formation (see above) and is known to be an important factor in maintaining seed and vegetative bud dormancy [91,92]. Turion formation and innate dormancy in the macrophytes *Utricularia macrorhiza* and *Myriophyllum verticillatum* were characterized by high endogenous levels of ABA, which decreased when the innate dormancy was lost [35]. Important for the maintenance of the innate dormancy of *S. polyrhiza* may be the high expression of genes encoding the LEA proteins that are known to promote and maintain seed dormancy [80]. The innate dormancy of the turion does not, however, completely exclude metabolic potential or activity. Dormant turions of *S. polyrhiza* have been observed to exhibit respiration [32] and to be capable of photosynthesis [32,49,93,94]. Dark respiration and photosynthetic capacity were likewise observed in dormant turions of other aquatic plants [95]. Innately dormant turions of *S. polyrhiza* were also observed to exhibit a slight gradual breakdown of storage starch [89], accompanied by a slight accumulation of low molecular weight carbohydrates [96].

The dormancy of newly formed turions is not due to insufficient nutrient reserves to fuel metabolism: turions of *S. polyrhiza* contain 60–70% starch in terms of dry weight [88,97,98]. The dormancy could, however, relate to a lack of availability of this polymeric carbohydrate reserve for turion metabolism. Freshly harvested *S. polyrhiza* turions indeed germinate to a considerable extent even without after-ripening when produced or kept in the presence of an external sugar supply [20,89,96]. Newly formed turions may thus not normally contain soluble, readily metabolizable carbohydrate sufficient to permit significant metabolic activity (see [96]), especially as such carbohydrate accounts for only about 3% of the total non-structural carbohydrate of the dormant turions [88].

#### 3.2.2. Innate Dormancy Becomes Imposed Dormancy

The winter cold that turions are exposed to for long periods under winter conditions is not only something to be endured, it is a prerequisite for the eventual activation of the dormant turions when conditions conducive to growth have set in. In nature, newly formed *S. polyrhiza* turions become able to germinate and resume normal vegetative growth only after prolonged exposure to cold, but not freezing temperatures (“chilling”) on the bottom of the water body to which they have sunken. This “after-ripening” takes place gradually: the length of the response has been observed in experimental investigations to depend on the conditions to which the turions have been subjected [26,33,36,37]. Several weeks at water temperatures of 0–5 °C are usually required to remove the innate dormancy of *S. polyrhiza* turions [33]; a period of 4–5 weeks is routinely used for experimentally breaking dormancy (see [51,94]). Due to the prolonged chilling required to break winter dormancy, turions do not germinate or sprout precociously before the cold season has passed and conditions have again become conducive to growth. The pre-winter formation of *S. polyrhiza* turions in response to nutrient deficiency may also take place while conditions are still warm and well-illuminated. If the turions were not dormant and did not require cold after-ripening to break the dormancy, the turions could germinate soon after their formation but not have sufficient time to grow and produce new turions in the face of imminent winter water surface conditions.

When turions have lost their dormancy through after-ripening, they can germinate, sprout and resume growth under appropriate conditions of temperature and light. However, they will not do so until these conditions are actually in place. If they are not, the after-ripened turions remain quiescent in “imposed” dormancy (able to germinate but prevented from doing so by environmental constraints). This imposed dormancy persists after completion of after-ripening on the bottom of the water body until the water temperature increases sufficiently to permit germination. The metabolic state of imposed dormancy resembles that of the resting fronds still capable of growth that lack true dormancy.

The loss of innate dormancy through after-ripening reflects the removal or counteracting of the metabolic block. This probably results from low metabolic activity present in the dormant turion. The dark respiration mentioned above to have been observed in innately dormant *S. polyrhiza* turions is evidence of such activity, and a progressive change in photosynthetic potential during after-ripening of *S. polyrhiza* turions [94] is an indication of structural/physiological modifications accompanying the breaking of the dormancy. Since newly formed turions of *S. polyrhiza* do not contain sufficient soluble, readily metabolizable carbohydrate for germination (see above), the gradual release from turion dormancy via after-ripening may be related to a gradual breakdown of polymeric starch molecules to soluble mono- or oligomeric carbohydrates which could eventually fuel germination metabolism. A gradual breakdown of the starch stored in newly formed *S. polyrhiza* turions that takes place upon extensive storage of the turions under cold aqueous conditions [89] is accompanied by a modest accumulation of soluble sugars [96]. The accumulation of this carbohydrate to an extent sufficient to enable germination may be dependent on low temperatures; under warmer conditions, the gradually accumulating carbohydrate might be consumed by an increased rate of basal turion metabolism.

Innate dormancy in turions can also be artificially broken to result in the ability to germinate. Experimental treatments with metabolic inhibitors such as potassium cyanide and 2,4-dinitrophenol, osmotic stressors such as polyethylene glycol, and phytohormones such as indole acetic acid and cytokinins have been shown to rapidly remove the innate dormancy of *S. polyrhiza* turions [26]. Such treatments apparently remove or counteract the metabolic block constituting innate dormancy and thus result in at best imposed dormancy and in the ability to germinate. It is notable that innate dormancy can even be prevented: turions produced by *S. polyrhiza* fronds growing in the presence of nickel are not innately dormant and can germinate as soon as after their formation and maturation is complete [56]. These turions thus exhibit at best imposed dormancy under conditions unsuitable for germination. The effect of nickel is thus to prevent the establishment of the metabolic block during turion formation and shows that metabolic quiescence is not obligately coupled to turion formation.

### 3.3. Turion Activation: The Resumption of Growth

Turions that have survived the cold of the winter and lost their innate dormancy on the bottom of water bodies must float up to the water surface in the spring to germinate and resume growth as “normal” fronds there.

#### 3.3.1. Turion Surfacing

After-ripened turions of S. *polyrhiza* surface rapidly under germination-inducing conditions; essentially all turions of control cultures reached the water surface within 48 h [99]. The actual mechanism of turion surfacing is not clear, but submerged turions of *S. polyrhiza* have been observed to expel a small bubble of gas upon light incidence when the water temperature increased to >15 °C. This bubble adheres to the junction between the pocket sheath and the upper surface of the turion and provides the turion with the buoyancy necessary to rise [36]. The gas of the bubble may be photosynthetic oxygen, as submerged turions have been shown to carry out photosynthesis and to surface at rates correlated positively with the rates of photosynthesis [93]. However, turion surfacing in *S. polyrhiza* after chilling has also been observed in the dark [20], in agreement with the fact that the turions often spend the winter under mud and litter at the bottom of their water body. Surfacing may also be encouraged by expanding intercellular gas spaces that have been described for turions of the carnivorous waterwheel plant *Aldrovanda vesiculosa* [100] and the water milfoil *Myriophyllum verticillatum* [101] and recently indicated by the analysis of submerged and floating *S. polyrhiza* turions by X-ray computed microtomography [102]. The bubble formation and turion surfacing are indicative of gas-producing metabolic activity accompanying the breaking of imposed dormancy coordinated with the onset of temperatures warm enough to support “normal” vegetative growth. An increase in nitrate reductase and nitrite reductase activities upon the warming of cold-after-ripened *S. polyrhiza* turions [103] is a further instance of this early post-dormancy metabolism. The presence of the heavy metals cadmium and nickel can slow turion surfacing in *S. polyrhiza* [99].

#### 3.3.2. Germination and Sprouting

The actual resumption of growth commences with germination. “Germination” is the onset of developmental processes in quiescent turions as observed in terms of the reflection of leaves or scales and a slight elongation of the internodes of primordial shoots emanating from the meristematic pockets of the turion [35]. The first indication of this in after-ripened *S. polyrhiza* turions is a slight swelling, after which 2 to 5 roots push through the root shield and the first primordial shoot pushes aside the pocket sheath as it emerges from the pocket. After a second primordial shoot develops, germination can be considered to have taken place [36]. Germination normally begins shortly after the turions reach the surface of the water and is dependent on temperatures of about 15 °C or higher and light [2]. As with turion surfacing, the presence of cadmium and nickel can slow *S. polyrhiza* turion germination but does not affect the vigour of the subsequent sprouting once germination has taken place [99].

Once *S. polyrhiza* turions have germinated, they “sprout” to resume vegetative growth, i.e., the production of new actively metabolizing and growing fronds. “Sprouting” commences with the distinct elongation of the still very short internodes of the germinated turions to enable better access to light, gas, and solute exchange for the emerging tissues, followed by the formation of new “normal” frond structures in the apical meristems (see [35]). Water temperatures favorable for germination (i.e., ≥15 °C) and light are key ecological requirements for turion sprouting. The presence of the numerous transcripts stored in the turions of *S. polyrhiza* as described in Sections “Transcriptome analyses of *S. polyrhiza* turion formation” and 3.3. promote both germination and sprouting when these have been initiated by enabling the rapid synthesis of required proteins.

The term “germination” is often used to encompass both “germination” and “sprouting” as outlined above (see [35]). However, analysis of the response of after-ripened *S. polyrhiza* turions to light has revealed that germination *per se* and its aftermath─sprouting─can indeed be separated and shown to be distinctly regulated aspects of an overall developmental process.

##### Light Dependence of Turion Germination

Light has long been known to trigger turion germination [2,36], and the germination response of surfaced *S. polyrhiza* turions to light is mediated by phytochrome. A single pulse of red light (“Rp”) induces germination effectively and can be reversed by a subsequent pulse of far-red light (“FRp”) [104]. This is a low fluence-type, “classical” phytochrome response [2,36,105]. Germination can also be induced to a similar extent by repeated red light pulses (“rRp”) or continuous red light (“cR”) [96,106], which indicates a special low-fluence response that requires newly formed phytochrome in its far-red light absorbing, physiologically active form over an extended period [97].

Light incidence on surfaced, after-ripened *S. polyrhiza* turions can trigger germination even when only a few of the turion cells are irradiated, whereby a local phytochrome-induced signal can be transmitted to the rest of the turion [107]. It took at least two days for turion germination resulting from Rp incidence to be observed [107], and longer response times may result from only partial irradiation as well as from physiological differences between turions.

##### Structural Changes Leading to Turion Germination

Light and electron microscopy of after-ripened *S. polyrhiza* turions exposed to irradiation with continuous red light have provided insight into the structural changes associated with germination [84]. Especially the two (frond) primordia of the no longer dormant turions were observed. The earliest cytological change observed was the formation of dictyosomes in the cells of these primordia. An increase in intercellular spaces, the disappearance of prolamellar bodies, and the formation of chloroplasts within the primordial cells were all observed within the first 48 h of illumination, after which visible germination became evident. The starch content of the chloroplasts of especially the apical cells of the primordia increased during the pre-germination period, indicating that carbohydrate was being translocated from the storage tissues of the turion to the frond primordia to fuel the elongation growth constituting visible germination.

##### Ion Requirements of Phytochrome-Mediated Turion Germination

The phytochrome-mediated germination of *S. polyrhiza* turions requires calcium [105], and the Ca^2+^-fluxes implicated may be related to changes in ion currents specifically observed upon light-mediated germination [108]. Whereas the phytochrome-mediated germination in *S. polyrhiza* turions elicited by a Rp does not take place in the absence of external Ca^2+^, it does upon irradiation with cR [106]. This shows that the “classical” phytochrome induction pathway has a different Ca^2+^ sensitivity than does the one requiring prolonged incubation with red light.

Phytochrome-mediated germination of cold after-ripened *S. polyrhiza* turions requires nitrate, not as a regulatory element in the signal transduction leading to germination, but rather as a prerequisite for the developmental process [109]. Nitrate (together with light) induces a coordinated synthesis of enzymes of nitrogen assimilation (nitrate reductase [110], nitrite reductase [103], glutamate synthase [111], and glutamine synthetase: [112]) parallel to germination. UVB irradiation was found to repress the light-stimulated induction of N-enzymes in *S. polyrhiza*, but not germination [113]. The regulation of germination and that of nitrate assimilation must thus be unrelated phenomena in the turions.

##### The Role of Starch Breakdown in Germination and Sprouting

Under natural conditions, germination is closely followed by sprouting, and the breakdown of the considerable reserves of starch stored in the turions (see [85]) would appear to be predestined to provide energy and carbon skeletons for the course of both developmental processes. Starch degradation in *S. polyrhiza* has been observed to progress extensively upon irradiation with continuous red light, but not in response to a red pulse [89,98]. Thus, only the low fluence phytochrome response requiring prolonged incidence of red light induces reserve starch degradation, whereas the classical phytochrome response gives rise to germination but not to starch degradation. This led to the conception that starch degradation does not contribute directly to germination, whereas it is closely associated with the subsequent growth of the newly produced shoots, i.e., sprouting [89,106]. Of course, sunlight in nature ensures both germination and starch degradation with its cR component.

If germination proceeds independently of starch degradation, what fuels the germination process? Although, as mentioned above in Section 3.2.1., freshly harvested turions of *S. polyrhiza* will normally not germinate in the presence of light, they will do so to a significant degree (about 50%) even in the absence of light when the medium is supplemented with sugar. This suggests that the inability of dormant turions to germinate is at least in part due to a lack of sufficient soluble, readily metabolizable carbohydrate. Measurements of the levels of soluble sugars in freshly harvested and cold after-ripened *S. polyrhiza* turions show that the after-ripened, non-dormant turions contained considerably more soluble sugar (approx. 190 µmol/g fresh weight (FW), as glucose, fructose, and maltose) than did the freshly harvested, dormant ones (55 µmol/g FW [96]). This accumulation of soluble sugar during the cold after-ripening of the dormant turions probably derives from the slow breakdown of some of the reserve starch of the turions that was observed to take place under cold storage conditions [89]. About three quarters of the soluble sugar having accumulated in the non-dormant turions was consumed independently of germination upon transfer from the cold after-ripening conditions (5 °C) to the 25 °C at which germination was tested (a further—see above—example of pre-germination metabolism induced in non-dormant turions by rising temperature). Germination was accordingly seen to correspond to a consumption of about 50 µmol sugar/g FW [96]. While this modest consumption is enough for germination to take place, it is not, as is discussed below, sufficient to support sprouting and thus ensure the success of germination.

Freshly germinated turions *S. polyrhiza* are already equipped with effective photosynthetic and respiratory machinery [32,94]. However, the assimilative potential of the newly sprouted fronds is limited. Although a single red-light pulse results in good germination of cold after-ripened *S. polyrhiza* turions, it leads to only very limited growth of the emergent sprouts. The weight of turions germinated in response to a Rp only doubled in the two weeks following the irradiation, whereas the growth of the newly emerging shoots progressed much more rapidly under cR irradiation [96]. This rapid sprouting is enabled by the breakdown of the reserve starch of the turions that is initiated by the cR treatment. The effect of cR in triggering *S. polyrhiza* turion starch breakdown is rapid and massive starch breakdown lags only about 12 h behind germination, with the starch reserves of the turion being exhausted within a week [106].

The rapid mobilization of turion storage starch in nature occasioned by the cR component of sunlight thus provides young fronds emerging from turions upon germination with a supply of readily metabolizable carbohydrate sufficient to support the rapid frond growth and development of sprouting. This, together with the early surfacing and germination of after-ripened turions, is propitious for enabling the newly formed fronds to occupy the water surface before other plants in the spring.

##### Mechanistic Aspects of Reserve Starch Degradation

As does the induction of germination by Rp, the induction of starch degradation in cold after-ripened *S. polyrhiza* turions by cR requires nitrate [114]. Additionally, as in the case with germination, the induction of the starch degradation is dependent on Ca^2+^ and subject to ion antagonism by Mg^2+^ [106,115]. Since the Ca^2+^ requirement of starch degradation is, however, far lower than that of germination, the Ca^2+^-sensitive steps in the two processes must be different [106].

The induction of *S. polyrhiza* turion starch degradation is associated with *de novo* protein synthesis [98]. Appenroth and co-workers showed that activity and protein of the starch-degrading enzyme β-amylase were induced via the classical phytochrome response parallel to germination [116]. However, a later study revealed that cR both inhibited the β-amylase induction by a Rp and resulted in a decrease in the endogenous activity of starch phosphorylase, another starch-degrading enzyme. Since the activities of the two enzymes did not correlate with starch degradation, they are most probably not causally involved in the mobilization of the turion reserve starch [117].

**Figure 4:**
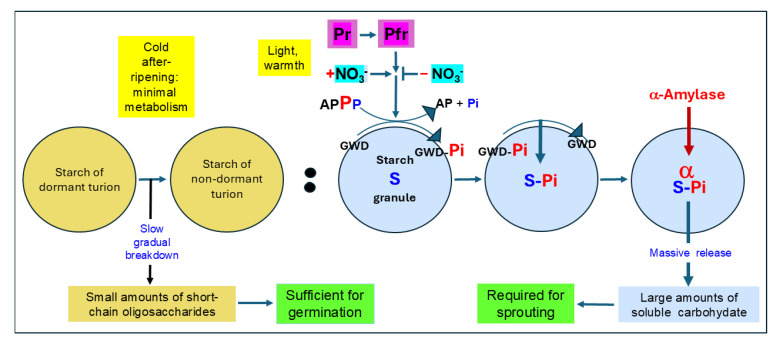
The fate of the starch stored in dormant turions of *S. polyrhiza*. The breaking of turion dormancy during the winter upon after-ripening is accompanied by the release of small amounts of soluble short-chain carbohydrates from the starch that accumulate sufficiently to support germination of the non-dormant turion. When germination occurs upon warming in the spring, incident light transforms the red light-absorbing form of phytochrome (“Pr”) in the emerging shoot into the far red-absorbing form (“Pfr”) that initiates the phosphorylation of glucan, water dikinase (“GWD”) at the starch granule surface. Phosphorylated GWD transfers its phosphate group to the starch granule itself, which enables α-amylase to bind to the granule surface and initiate massive starch breakdown to release large amounts of soluble carbohydrates to support sprouting.

More recently, the enzyme α-amylase, together with a protein dubbed “R1”, was found to be bound to the reserve starch granules of cold after-ripened, dark-adapted turions of *S. polyrhiza*. The extent of binding increased transiently upon cR-induced starch degradation and decreased strongly once starch degradation commenced [118]. α-Amylase is considered to initiate the degradation of reserve starch in a “pace-maker” mode in tissues such as cereal seed endosperm in which the starch grains are not surrounded by intact amyloplast membranes [119,120,121]; (see also [122]), but its role in starch degradation in intact plastids of leaves is not so clear and appears to be of rather minor importance [120,123,124]. The “R1” protein was revealed to be the starch-phosphorylating protein glucan, water dikinase (GWD), an enzyme essential for starch degradation [125,126]. The same light signal that induces starch degradation in cold after-ripened *S. polyrhiza* turions (cR) brought about the auto-phosphorylation of starch-associated GWD, which is thought to lead to starch breakdown via transfer of the phosphate group to the starch granule surface [127]. Irradiation of the turions with cR indeed resulted in the phosphorylation of the turion starch and in a transitory enhanced binding of α-amylase to the starch; the α-amylase appeared to be actively involved in degrading the starch [122]: see Figure 4. Electron microscopy studies of starch granules during cR-induced starch degradation revealed surface erosion of the starch grains as well as the formation of tunnel-like erosion channels within the granules. In addition, evidence was presented for the integrity of the amyloplast membranes enclosing the starch grains throughout the degradation [85]. Together, these findings point to starch degradation in *S. polyrhiza* turions taking place via the activity of α-amylase in intact amyloplasts. This indicates that *S. polyrhiza* turions exhibit a unique system of starch degradation that combines the intact plastids known from leaves with the action of α-amylase known otherwise to effect starch breakdown in non-compartmented endosperm environments. The starch degradation in the turions is thus remarkable not only for the means of its induction (by light and nitrate), but also for the means of its realization (see also [128]).

## 4. The Turion Biology of *S. polyrhiza*: A Good Model for Turion-Based Duckweed Overwintering?

The formation, dormancy, and activation of turions in the life cycle of *S. polyrhiza* constitutes an effective and elegant strategy for surviving severe winter conditions in an aquatic environment in a vegetative mode by the avoidance of lethal cold: this is particularly well illustrated by the transcriptome study of Pasaribu and co-workers [81]. Since turion formation also occurs in other duckweeds, can the information on the developmental cycle of turions in *S. polyrhiza* as described in this article serve as a model for understanding turion-based duckweed overwintering overall? Is this information pertinent to the understanding of the overwintering of all duckweed resting fronds, since turion-like resting fronds share some functional equivalence to true turions?

Duckweeds are suitable as model organisms for the investigation of many aspects of plant biology because they on the one hand are amenable to the collection of pertinent experimental data on account of their small size, simple structure, rapid growth, and ease of cultivation. They have accordingly been extensively researched: well-developed experimental protocols and molecular tools are available for their investigation, and they are of interest for an international community of experts represented by the International Steering Committee on Duckweed Research and Applications [4]; see [129]. Indeed, duckweeds are regarded as model organisms to investigate aquatic toxicology of higher plants [16], and they have been and are useful in studies on plant biochemistry and physiology [17], ecology and evolution [129,130], and multiple additional topics of modern plant biology [4,131]. *S. polyrhiza* itself has been regarded as a model organism for studying dormant bud induction [66] and low-fluence phytochrome responses in plant development [97], and even its turions are seen as model objects in ecotoxicological testing [132].

A model organism is a representative of a group of organisms exhibiting certain common features of interest. It is chosen to provide information on these features that should apply to all organisms of the group. If information obtained from a putative model organism is found to be valid for the other members of the group, then this organism is a good model for the subject in question. In this case, the model organism is truly representative of the group and information obtained for it can be assumed to be relevant to the other group members. If investigation of the representative organism yields information that is not or only partially valid for the other members of the group in question, then no satisfactory model role can be ascribed to this organism. The putative model organism then represents rather an exceptional member of the group. Judgement as to whether a putative model organism is a good one or not is dependent on the availability of data sufficient for making the judgment. If these data are not available, an organism selected for a model role can be used as reference for the collection of comparative information on the other members of its group. This may eventually result in the chosen organism exerting a model role in the full sense.

If *S. polyrhiza* is to be a good model species for investigating turion-based duckweed overwintering, its cycle of turion formation, dormancy, and activation must be shown to correspond logistically and functionally in an at least overall sense to equivalent cycles in the other turion-producing duckweeds. If it is to be a useful model species in a wider sense of investigating resting frond-based duckweed overwintering, then its turion developmental cycle should also have significant aspects in common with the formation, quiescence, and activation of the duckweed resting fronds still capable of growth.

### 4.1. Other Duckweed Turions in Relation to S. polyrhiza

As indicated in Section 2.2., true turions are found in *Le. turionifera*, *Le. aequinoctialis,* and many species of *Wolffia* as well as in *S. polyrhiza*.

The induction of turion formation in the two *Lemna* species can be only very incompletely related to what is known for *S. polyrhiza*. *Le. turionifera* formed turions upon the exogenous addition of ABA does as *S. polyrhiza* [133], a response that has been used as a positive control for the induction of turion formation [134]. Although nutrient─and especially phosphorus─limitations were reported to induce turion formation in *L. turionifera* [135], another study concluded that nitrogen or phosphorus deficiency─which is the prime natural factor leading to turion formation in *S. polyrhiza*─was ineffective in inducing turion formation in this species, whereas supplementation with sucrose did lead to turion formation [136]. There was no mention of turion formation in *Le. turionifera* grown under nitrate or sulphur deficiency [137,138] or in *Le. aequinoctialis* grown under nitrate starvation [139], i.e., under conditions resulting in turion formation in *S. polyrhiza*. There are no reports as to the structure, dormancy, or activation of either *Le. turionifera* or *Le. aequinoctialis* turions.

There is very little information on the structure or physiology of *Wolffia* turions [31]. *Wo. arrhiza* turions were described as being smaller and more simply formed and exhibiting smaller airspaces and much more starch than the mother fronds [139]. Their formation could be interpreted to reflect mineral nutrient deficiency and was enhanced by added sugar [140], and turions were also found to be produced in *Wo. arrhiza* under nutrient deficiency [141,142]. These findings are similar to those respective of *S. polyrhiza* structure and formation, but the mature turions of *Wo. arrhiza* were not dormant and could germinate rapidly [140]. Turion formation in *Wo. brasiliensis* appeared to be more independent of low nitrate or phosphate concentrations than in *S. polyrhiza* [143]. There are no reports on *Wolffia* turion dormancy or activation.

Turions described for *Wa. floridana* (now *Wa. gladiata)* were somewhat shorter and wider than the mother fronds; they contained smaller air spaces and much more starch than did the fronds and sank to the water bottom upon maturation [30]. Although these features indicate similarity to *S. polyrhiza* turions, the developmental characteristics of the *Wa. floridana* turions were quite different than those of *S. polyrhiza*. The formation of the turions could not be induced by ABA application, but rather upon increasing the sucrose content of the medium from 1% to 3%. The turions were also not truly dormant: they germinated and sprouted within a week after transfer to medium with 1% sucrose. There is no information as to their activation.

### 4.2. Other Duckweed Turion-Like Fronds in Relation to S. polyrhiza

As indicated in Section 2.1, *La. punctata*, *Le. perpusilla*, *Le. gibba*, *Le. minor*, *Le. aequinoctialis*, and *Le. japonica* form resting fronds capable of growth that remain on the water surface, whereas equivalent resting fronds of *Le. trisulca*, *Wa. gladiata*, and *Wo. arrhiza* sink to the bottom of the water body.

Turion-like resting fronds were formed during the early winter in *Le. aequinoctialis* (referred to as *Le. paucicostata*). They featured, as in *S. polyrhiza*, much smaller air spaces and more starch than the mother fronds and spent the winter at the bottom of the water container [144]. Non-dormant resting fronds of *Le. minor* were observed to have open stomata illustrating growth potential [64]. As with *S. polyrhiza*, the application of ABA, as well as its allenic analogues, induced the formation of turion-like resting fronds in *Le. aequinoctialis* (referred to as *Le. paucicostata*) and *Le. gibba* [145,146]. ABA was also reported to induce resting frond formation in *La. punctata* (referred to as *S. punctata*), and *Le. gibba* [26], and to severely inhibit the growth of *Le. minor* fronds [147]. These findings suggest that ABA may be involved in the formation of all duckweed resting fronds (see [64]). The formation of *Le. minor* “turions” was, as in the case of *Le. turionifera* and in contrast to *S. polyrhia*, due rather to supplementation with carbohydrate than to mineral nutrient deficiency [136]. The formation of resting fronds in *Le. perpusilla* was induced by short days under the control of phytochrome [148], and long days were required for resting fronds in *La. punctata* (referred to as *S. punctata*: [2]).

### 4.3. Conclusions and Outlook

These few findings thus show individual indications of correspondence with principles underlying the induction of turion formation of *S. polyrhiza* (induction by ABA application and by mineral salt and especially phosphate deficiency, carbohydrate supplements only enhancing induction, and lack of photoperiod control) in other turions or turion-like resting fronds. In some cases, however, examples contradictory to these principles are evident (lack of induction by ABA or phosphate/nitrate limitation, sugar supplementation being the inductive factor itself, and induction by long or short days). It cannot be concluded from this very limited information that the *S. polyrhiza* model of turion formation, structure, dormancy, and activation is satisfactorily applicable to either the other (i.e., non-*S. polyrhiza*) duckweed turions or to duckweed resting fronds on the whole.

The main factor determining this insufficiency is the widespread lack of knowledge about the other duckweed turions/turion-like resting fronds. The turion biology of all other appropriate duckweed species has been investigated at most cursorily, never to an extent at all comparable to that of *S. polyrhiza*, and often not at all. The turion biology of *S. polyrhiza* can thus serve as a model for turion- (or resting frond-) based duckweed overwintering in the sense of being a reference point for corresponding data to be obtained from other turion- or resting frond-bearing duckweed species. The study of these other species must encompass the ultrastructure of the respective resting fronds, the identification of the factors that induce and promote the formation of the resting fronds, the determination of dormancy and─if appropriate─after-ripening, and the factors responsible for the germination or at least growth resumption of the resting fronds. It would be of advantage to follow standardized procedures to obtain the required comparative information. The sufficient characterization of the species producing true turion and turion-like fronds in these regards would enable a confident judgement as to whether the turion biology of *S. polyrhiza* is indeed representative of the resting frond biology of these species and thus a good model system for further in-depth analysis of turion- or resting frond-based duckweed overwintering.
